# Marine-Derived Lipases for Enhancing Enrichment of Very-Long-Chain Polyunsaturated Fatty Acids with Reference to Omega-3 Fatty Acids

**DOI:** 10.3390/md22070301

**Published:** 2024-06-28

**Authors:** Mahejbin Karia, Mona Kaspal, Mariam Alhattab, Munish Puri

**Affiliations:** 1 Bioprocessing Laboratory, Medical Biotechnology, College of Medicine and Public Health, Flinders University, Bedford Park, Adelaide 5042, Australia; 2 Flinders Health and Medical Research Institute, Flinders University, Bedford Park, Adelaide 5042, Australia

**Keywords:** lipase, polyunsaturated fatty acids, omega-3 enrichment, marine lipase, immobilization

## Abstract

Omega-3 fatty acids are essential fatty acids that are not synthesised by the human body and have been linked with the prevention of chronic illnesses such as cardiovascular and neurodegenerative diseases. However, the current dietary habits of the majority of the population include lower omega-3 content compared to omega-6, which does not promote good health. To overcome this, pharmaceutical and nutraceutical companies aim to produce omega-3-fortified foods. For this purpose, various approaches have been employed to obtain omega-3 concentrates from sources such as fish and algal oil with higher amounts of eicosapentaenoic acid (EPA) and docosahexaenoic acid (DHA). Among these techniques, enzymatic enrichment using lipase enzymes has gained tremendous interest as it is low in capital cost and simple in operation. Microorganism-derived lipases are preferred as they are easily produced due to their higher growth rate, and they hold the ability to be manipulated using genetic modification. This review aims to highlight the recent studies that have been carried out using marine lipases for the enrichment of omega-3, to provide insight into future directions. Overall, the covalent bond-based lipase immobilization to various support materials appears most promising; however, greener and less expensive options need to be strengthened.

## 1. Introduction

Marine environments cover the majority (70%) of the total Earth’s biosphere, representing an exhaustive and wide range of diverse groups of flora and fauna that have developed a unique way to adapt to extreme environmental conditions, producing novel metabolites [1]. As such, marine ecosystems are considered a renewable resource for various commercially valuable components such as enzymes, vitamins, antibiotics, drugs, bio-emulsifiers, biosurfactants, and biofuels, which have significant applications in biotechnology and biomedical industries [2,3,4]. Similarly, the biomolecules, carotenoids, lipids, saponins, phenolics, and polysaccharides obtained from marine sources also have great biological value in functional foods and nutraceuticals [5]. In particular, over the past few decades, marine microorganisms have attracted attention towards unexplored marine enzymes and their application in food processing as they are more stable compared to those from animal and plant origins [2,5]. Various studies have identified the special function of lipase enzymes from marine fungi, bacteria, actinomycetes and other microorganisms, which have been utilised in different industries such as leather, textile, pharmaceuticals, food, biodiesel, agrochemical, and cosmetic industries due to their properties and ability to catalyse various biotechnological reactions, and the diversity of microbial, animal, and plant genes that encode lipases [6,7,8,9,10]. More recently, the use of lipases has proven effective in concentrating polyunsaturated fatty acids (PUFAs) [11], which is significant as the global demand for essential omega-3 was valued at USD 7.5 billion in 2024 and is expected to increase to USD 14.1 billion in 2029 [12] as a result of increased health awareness and associated health benefits.

Omega-3 and omega-6 fatty acids are the two major classes of PUFAs, which are named based on the position of the first double bond from the methylated end of the fatty acid chain [13,14]. Omega-3 PUFAs are considered essential fatty acids as they cannot be synthesised in the human body or that of animals [14,15] and must be consumed [16]. Recently, the change in diets consisting of increased fast-food intake has overturned the balance and dramatically increased saturated fatty acid consumption as compared to PUFAs. The low intake of essential dietary PUFAs is thought to be one of the main reasons for the increased risk of cardiovascular diseases, Alzheimer’s, and depression [17,18]. Omega-3 PUFAs have a vital role in maintaining the overall health and wellbeing of the human body and in reducing the risk of diseases [19]. Among the essential fatty acids, alpha-linolenic acid (ALA, 18:3, n-3), linoleic acid (18:2, n-6), and Docosahexaenoic acid (DHA, 22:6, n-3) are very important polyunsaturated fatty acids for maintaining functions in mammals [20]. Research performed using alpha-linolenic acid showed that an increased dietary intake of ALA assisted in reducing blood pressure [21]. In addition, an epidemiological study carried out in 2004 suggested that fish oil supplemented with omega-3 (DHA and EPA) demonstrated potential in reducing the risk of heart attacks and cardiovascular-related deaths [22]. Omega-3 supplementation in diets has also been investigated for minimizing the possibility and treatment of breast cancer in women from different countries [23]. Furthermore, the various health benefits of dietary intake of omega-3 fatty acids from different sources (plants, fish, and microbial)—relating to cardiovascular diseases, hormonal balance, inflammatory responses in different developmental stages of life, and neurocognitive and visual development in the early stages—have been discussed by Calder [24]. As such, the regulation of incorporating DHA in infant foods was made compulsory in almost every country of the world [25,26].

Furthermore, the health benefits associated with omega-3 PUFAs, and the low quantities available in diets consumed today, have increased the demand for functional foods’. This has elevated the trend of food fortification, adding small quantities of omega-3 fatty acids in dairy, baked goods, dressings, spreads, meat products, and chocolates [27]. As such, nutraceutical and food industries have become more interested in developing omega-3-enriched foods, which require omega-3 PUFAs in a concentrated form. There are various strategies such as molecular distillation, urea complexation, supercritical fluid extraction, and enzymatic enrichment employed to concentrate omega-3 PUFAs [28,29,30,31].

Among these, enzymatic enrichment is a highly studied alternative as it requires less energy and can be performed at lower temperatures, which prevents omega-3 oxidation and degradation [28]. Marine lipases are increasingly used for this purpose as they possess fatty acid selectivity that allows them to hydrolyse saturated and monounsaturated fatty acids [32]. However, the major drawback of using free enzymes is the loss of enzymatic activity and stability due to denaturation and its limited reuse. Marine microorganisms have been considered recently as preferred sources for lipase extraction due to their specific characteristics; however, further studies are required to identify if lipases from marine microorganisms contain any specific characteristics such as improved stability and ease of large-scale production [8]. For enhancing the stability and activity of enzymes, immobilization techniques can be carried out [33,34,35]. The aim of this review is to provide inference on the recent advancements made in utilising marine lipases as an effective means for enriching omega-3 PUFAs, and future research directions.

## 2. Lipases and Their Sources

**Figure 1:**
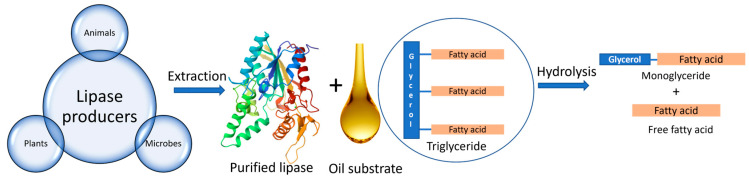
Various sources of lipase enzyme and its mechanism of action.

Lipases are basically triacylglycerol ester hydrolases that have the ability to hydrolyse fats and oils [36]. Lipases cleave ester bonds present in triglycerides to form monoglycerides and free fatty acids [37], as seen in Figure 1. Lipases can also catalyse other types of reactions such as esterification, transesterification, interesterification, and amino lysis [38]. Their molecular size ranges between 20 and 60 kDa and comprises 270 to 641 amino acids [38]. Lipases possess a unique property of interfacial activation, which allows the catalysis of lipids at the lipid–water interface. Lipases contain a helical oligopeptide unit referred to as Lid, which assists in activating the active site of the enzyme under specific conditions such as in the presence of micellar substrates [39]. Moreover, the specificity of lipases also depends on the size and hydrophobicity of the catalytic beads. The active site comprises a catalytic triad of three amino acids: serine, histidine, and aspartate [40]. In the active site, there are four substrate-binding pockets for triglycerides that can accommodate fatty acids at the sn-1, sn-2, and sn-3 positions [38]. The selectivity of the lipases can be enhanced by using acylating agents, organic solvents, and additives, such as ethanol, and by changing the operating conditions such as temperature [41]. Lipases can be obtained from various sources such as plants, animals, and microorganisms.

### 2.1. Plant Lipases

Plant lipases are mostly present in seeds like sunflower, castor bean, almond, black cumin, and sesame [42]; moreover, fruit waste from orange, mango, papaya, and palm are also reported as good sources for lipase [43]. Plant lipases are used for various pharmaceutical purposes, such as *Carica papaya* latex, which is used in the production of canola phylosterol oleate esters that can be further utilised as cholesterol-lowering agents to reduce the risk of coronary disease [44]. However, there is a limitation of lower production of enzymes from plants, which makes purification procedures complicated and prone to activity loss [43].

### 2.2. Animal Lipases

Animal-derived lipase sources include mammals, insects, and fish. Animals like pigs, cattle, hogs, and sheep are used to extract pancreatic and progastric lipases [45]. Animal-sourced lipases such as pancreatic lipase have been extensively used for catalysing primary alcohol esters hydrolysis [46]. The lipases from animals were also used in the dairy industry for developing flavour in cheese and other products. For this reason, lipases from porcine pancreas have been used to induce flavour in cheddar cheese [47]. However, due to its low stability and complex recovery procedures, its use at a commercial level has been limited [48,49].

### 2.3. Marine Lipases from Various Microorganisms

Microorganism-based lipases from bacteria and fungi (Table 1) have wider applications in industry and are the most studied source of lipases due to their variety of catalytic specificity, simple genetic modifications, high growth rate, and the ability to grow in laboratory-controlled conditions [50]. The first microbial lipases were identified in bacteria *Bacillus prodigiosus*, *B.pyococyaneus*, and *B.fluoroscens* in 1901 [51]. Later on, various fungi and yeast were also found to synthesise lipases [52]. Microorganism-based lipases hold great commercial value due to their better stability, higher selectivity, and broad substrate specificity [53].

#### 2.3.1. Microbial Lipases

Marine microbes have recently achieved increasing attention as a source of bioactive metabolites that have various biomedical potential [74]. Marine microbes have the ability to adapt to various extreme environmental conditions and possess high genetic plasticity that can positively influence compound and secondary metabolite production [75]. Various marine bacteria and yeasts are used to extract cytotoxic compounds, enzyme inhibitors, and anti-inflammatory agents which have broad clinical importance [76].

The marine yeasts, *Candida antartica* and *Candida rugosa*, are the most widely used sources of lipases and are categorised as GRAS (generally regarded as safe). The GRAS status makes these lipases more suitable for various applications in food and clinical industries, such as flavouring agents and for the production of antioxidants [77]. Nine yeast strains—*Candida intermedia* YA01a, *Pichia guilliermondii* N12c, *Candida parapsilosis* 3eA2, *Lodderomyces elongisporus* YF12c, *Candida quercitrusa* JHSb, *Candia rugosa* wl8, *Yarrowia lipolytica* N9a, *Rhodotorula mucilaginosa* L10-2, and *Aureobasidium pullulans* HN2.3—were identified to produce stable lipases in the pH range of 6.0 to 8.5 and temperature range of 35–40 °C by Wang et al. [54]. *C. rugosa* lipases are sold by companies such as Sigma, Roche, and Amano in immobilized and lyophilic powder forms [78]. Various studies are carried out to improve the thermostability and activity of yeast-based lipases through immobilization, medium engineering, and protein engineering approaches [79,80]. The unique property of these lipases is their broad specificity towards long-chain triacylglycerols as they can hydrolyse shorter fatty acids at faster rates; therefore, they can be used for the enrichment of long-chain fatty acids [81].

Marine microorganisms are reported to have the ability to produce lipases with a varying range of lipolytic activity (Table 1). The marine bacteria, *Oceanobacillus caeni*, isolated from the east coast of India, has the unique property of being stable at a wide range of pH from 3 to 11 and temperatures of 10 to 70 °C [67]. Moreover, lipases from *Bacillus sonorensis* were studied for their efficacy as a detergent additive for the efficient removal of corn oil stains, where it was found that it was stable at a temperature ranging from 23 to 60 °C [68]. Furthermore, lipase from *Bacillus pumilus* B106, associated with the South China Sea Sponge *Halichondria rugosa,* has the appealing feature of tolerance towards high salinity, which is considered an important factor in producing biodiesel derived from marine organisms [82]. These studies suggest increased interest in lipase-producing marine bacteria in various industrial applications.

#### 2.3.2. Microalgae Lipases

There are various other studies that provide evidence of marine microalgae as lipase producers. A genomic study performed on the microalgae *Chlamydomonas reinhardtii* and *Chlorella vulgaris* revealed that they have lipase-encoding genes. In the case of *C. reinhardtii*, the galactoglycerolipid lipase-encoding gene CrLIP1 was identified using *E. coli* as a protein expression system and was obtained in purified form [83]. While *Chlorella vulgaris* demonstrated a total of 14 lipase-encoding genes, characterised using sequence homologies and bioinformatics tools, further attempts to extract and purify the lipases were not performed [84]. Moreover, Savvidou et al. [72] confirmed the presence of thermostable lipase enzymes in *Nannochloropsis oceanica* for the first time and were successful in extracting the lipases from the cell surface and cell debris fraction [72]. Similarly, extracellular lipases produced from *Botryococcus sudeticus*, a phototrophic microalgae, were purified and reported to exhibit various properties such as resistance towards a broad range of temperatures, pH tolerance, and showed variation in specificities for different substrates [73]. However, the presence of easily available commercial lipases on the market has limited investigations into microalgae-based lipase sources [84].

## 3. Lipase Commercial Market and Applications

### 3.1. The Lipase Commercial Market

After protease and carbohydrates, lipases make up the third-largest group of enzymes, based on their market value [85,86,87]. In 2019, the global market size for microbial lipases was valued at USD 349.8 million and is expected to maintain a compound annual growth rate (CARG) of 5.2%, reaching USD 428.6 million by 2025 [88]. This demonstrates the increased demand for lipases globally, and according to a review performed in 2021, the animal- and microbial-based lipase segments in 2020 held the largest market shares of 26.6% and 61.64%, respectively [89]. As of 2021, North America was the largest market producer of lipases, making up 38% of the total shares worth USD 263 million, followed by Europe, which accounted for 31% of global market share [90].

### 3.2. Marine Lipase Applications

The marine ecosystem is one of the major sources of biodiversity, especially due to the harsh conditions underwater, and various microorganisms have unique and sophisticated genetics and characteristics with the ability to produce bioactive compounds [91]. Recently, marine fungi have gained increased attention for lipase production, as they produce extracellular lipases, which have reduced fat and oil contents by 92% in oil-polluted effluent [86]. In 2014, alkaline-stable lipase enzymes were used in milk flavour production [92]. They are also used in the acceleration of cheese ripening, to modify flavours in food through the synthesis of ester short-chain fatty acids and alcohols [7,93]. They are used in food industries to process food such as fruits, meat, beer, and milk products, and to improve the flavour of dairy products [94]. Similarly, lipases are used in the textile industry, to help in the removal of lubricants to provide a fabric with high absorbency for improved dyeing [95]. They are also used for the production of paper and pulp by hydrolysing the wood triglycerides or waxes [93]. Lipases have their importance in biofuel production by transesterification of fats and vegetable oils with short alcohol chains [85]. Lipases are environmentally friendly as they can be used in detergents that allow lower wash temperatures with less toxic residues, fewer chemicals in the detergents, no threat to aquatic life, and no adverse effects on wastewater [96]. In addition, lipase solvents or detergents remove fats and greases from leather, which makes it soft and easy to use for further processing [97]. Furthermore, lipases also play a role in the pharmaceutical industry as they have the ability to prevent epimerization, rearrangement, racemization, and isomerization [96,98]. They also have several applications in the medical industry and have been utilised for their therapeutic and diagnostic uses in digestive tract disorder, pancreatic damage, and as a measuring tool for serum lipid profiles [97].

## 4. Omega-3 Enrichment Techniques

There are various methods used for concentrating omega-3 PUFAs from fish and algal oils. Most industries use fish oils like sardines to obtain omega-3 PUFA concentrates. Before concentrating omega-3 PUFAs at an industrial level, fat-soluble contaminants are removed by either adsorption processes or chromatographic methods [99]. This is then followed by the removal of the glycerol backbone in the presence of an alkaline catalyst to convert it into ethyl esters or free fatty acids. After removing the glycerol backbone, the enrichment process can be carried out using urea precipitation, supercritical fluid extraction, molecular distillation, and enzymatic enrichment, as seen in Table 2.

### 4.1. Urea Precipitation

Urea precipitation is a method that is based on the property of urea crystals to form complexes with straight-chain and monounsaturated fatty acids (MUFAs). To achieve this, urea is first dissolved into an organic solvent such as methanol or ethanol, which is allowed to cool down in the presence of decontaminated oil. While cooling, the formation of urea crystals occurs, which in turn traps the saturated and monounsaturated fatty acids allowing for the separation of PUFAs. Urea can be filtered to obtain a concentrated form of PUFAs [103]. This process can be helpful in obtaining omega-3 PUFA concentrates with 45–60% EPA (Eicosapetanoic acid) plus DHA (Docosahexanoic caid) content; however, the major bottleneck of this process is dealing with flammable solvents in larger volumes and the disposal of urea saturated with fatty acids, which make it a highly expensive process [99].

### 4.2. Supercritical Fluid Extraction

This method is primarily known for its ability to selectively separate Methyl stearate (C18), Methyl eicosapentanoate (C20), and Methyl docosahexanoate (C22) fatty acids using the supercritical form of carbon dioxide (SC-CO_2_), which can easily solubilize fatty acids with low molecular weight [104]. The additional and most important advantage of this technique is that SC-CO_2_ is non-toxic, non-flammable, and a clean solvent alternative [105]. DHA with high purity can be enriched at a ratio of 60% using this method [106]; however, carbon dioxide needs to be compressed to a pressure of more than 73 bar and temperatures of 32 °C to reach the supercritical state [100]. Moreover, SC-CO_2_ is pumped through a vertical column from the bottom to the top under high pressure, along with the fish oil ethyl ester continuously in the counter-current direction of the CO_2_. Thus, the major drawback of this technique is the high capital cost associated with the pressure-generating equipment [99].

### 4.3. Molecular Distillation

Molecular distillation is the most commonly used technique in industry nowadays to obtain concentrated omega-3 PUFAs. This method is based on the principle of differences in molecular weight and boiling point of the different fatty acids under low pressure [32]. At lower pressures and higher temperatures ranging from 140 to 170 °C, EPA and DHA content can be enriched by up to 60%, as compared to the original content of 30% in fish oil; however, the high temperatures used in this technique lead to the loss of fatty acid saturation and causes hydrolysis, isomerization, and thermal degradation of omega-3 PUFAs at certain levels [101].

### 4.4. Enzymatic Enrichment Method

The utilisation of enzymes at an industrial level is a recent and more advantageous option compared to other alternatives discussed above due to its simplicity in processing and low capital requirements; moreover, it eliminates the need for using organic solvents and high temperatures that can deteriorate the quality of omega-3 PUFAs. These positive aspects of enzymatic enrichment have promoted the interest of researchers to make this technology more efficient by developing different strategies. The use of enzymes such as protease, exopeptidase, endopeptidase, and lipase has shown better enrichment of omega-3 PUFAs [102]. Among these, lipases can catalyse hydrolysis and esterification reactions with high selectivity towards omega-3 PUFAs. The selectivity of the lipases is due to the greater number of double bonds present in EPA and DHA, which cause steric hindernce that acts as a barrier for the enzyme denying the hydrolyses of these fatty acids [107]. Thus, using lipase-based hydrolysis, free-form fatty acids, monoacylglycerols, diacyglycerols, and triacylglycerols are formed, which can be further separated by solvent extraction method [108]. This property of lipase enzymes makes them a highly suitable alternative for omega-3 PUFA enrichment through a two-step enzymatic method; however, the major drawback is the enzymes’ nature of getting easily denatured and their limited reusability. To mitigate these problems, intensive research has been carried out by researchers to develop different immobilization strategies to improve the stability, activity, and reusability of lipase enzymes [109] (Section 5).

## 5. Marine Lipases in Enriching Omega-3

Lipases have the ability to selectively enrich EPA and DHA content as they have a partial selective ability towards chain length, position of the fatty acids, and *cis*-double bonds [110]. Conventionally, ethanol-based transesterification was used at an industrial level to produce concentrated PUFA esters [111]; however, this type of chemical transesterification can lead to deteriorating effects on the oxidative stability of oil [112]. Recently, various studies have carried out the enrichment of EPA and DHA from different oil sources, with the majority being fish oil using lipases, see Table 3.

In a study conducted by Yang et al. [113], the commercially available lipases AY “Amano” 400SD (free lipase from *Candida cylindracea*, 400 U/mg), AY “Amano” 30SD (free lipase from *Candida cylindracea*, 30 U/mg), AY “Amano” S (free lipase from *Candida cylindracea,* 30 U/mg), DF “Amano” 15 (free lipase from *Rhizopus oryzea*, 150 U/mg), G “Amano” 50 (free lipase from Aspergillus oryzae, 50 U/mg), and nonspecific Lipozyme 435 (immobilized lipase from *Candida antarctica*, 10 U/mg, particle form) were tested for their omega-3 enrichment specificity using tuna oil. It was concluded that AY “Amano” 400SD possesses the most effective selectivity to hydrolyse saturated fatty acids and monounsaturated fatty acids by increasing the omega-3 PUFA content from 34.3% to 57.7%, while the other lipases accounted for PUFA content increases from 34.3% to less than 50% [113].

Lipases from marine natural sources are also extensively studied for their enrichment ability. For instance, in the study of Baloch et al. [114], lipases were produced from three different yeast strains *C. rugosa* TISTR 5627, *Y. lipolytica* TISTR 5212, and *P. guilliermondii* TISTR 5142 and a comparison of their ability to concentrate EPA and DHA was made; it was reported that *C. rugosa* lipase had better enrichment ability compared to the lipases from the other two strains [114].

Interestingly, various researchers have also attempted to produce recombinant lipase with better properties compared to lipases obtained from natural sources. In a recent study, a recombinant LipB from *Pseudomonas fluorescens* was cloned into *Bacillus subtillis*. It was reported that the recombinant lipase had excellent properties such as solvent tolerance against acetonitrile, isopropanol, acetone, and DMSO; moreover, it was able to increase the PUFA concentration from 43.2% to 72.2% using fish oil and had thermostability at 70 °C [115].

The study of López et al. [11] investigated the omega-3 enrichment of microalgal oil from *Nannochloropsis* species using the commercial lipases Lipozyme TL 100 L, Lecitase^®^ Ultra, Lipozyme^®^ CALB, Quara^®^ LowP, Lipase D, Lipase DF, Lipase MER, Lipase AY, and Lipase QLM^®^. It was found that the QLM lipase, extracted from *Alcaligenes* species, was highly efficient in enriching EPA polar lipids as it exhibited 1,3 positional specificity and this lipase was further used for a selecting solvent system. Moreover, lipase QLM was immobilized on Accurel MP 1000 and was able to enrich EPA from 48.5% to 70% with a recovery rate of 92% [11]. This study suggests that enzyme immobilization can be used to reduce the cost of the process by recovering and reusing the enzyme.

Lipases can also be used for enriching PUFAs in fortified oils. The research group at Deakin University used pure omega-3 ethyl esters that were then reacted with commercially available immobilized lipases from *Rhizomucor miehei* (Lipozyme RMIM), *Thermomyces lanuginosus* (Lipozyme TLIM), and *Candida antarctica B* (CALB, Novozym^®^ 435), and the monoacylglycerols and diacyglycerols obtained as products after the lipase Novozyme 435-mediated glycerolysis reaction were further fortified with extra virgin olive oil. The resulting fortified oil treated with lipase accounted for an increase in EPA content from 0.75% to 13% and a DHA content of 16% [116]. Novozyme 435 was also used for producing glycerides containing concentrated levels of omega-3 PUFAs, and it was reported that the synthesised glycerides had 1.21 and 2.71 times more EPA and DHA, respectively, as compared to the crude fish oil [117]. Moreover, Novozyme 435’s ability to catalyse acidolysis to produce DHA/EPA ethyl esters was also evaluated and analysed, and it was found that the enzyme was able to produce concentrated DHA/EPA ethyl esters with a 94% conversion yield ratio [118].

These studies demonstrate the ability of marine lipases to enrich omega-3 essential fatty acids using a simple process. The only bottlenecks are the risk of losing the lipase’s stability and activity after a single use, and the recovery of the free form of lipases is difficult to achieve. These constraints result in increased overall costs for producing enriched omega-3 concentrates. To mitigate this, lipases have been employed in their immobilized form in order to enhance reusability and recovery; moreover, the immobilization of lipases also tends to improve their overall efficiency [119].

## 6. Enzyme Immobilization for Advancing the Enrichment of Omega-3 Fatty Acids

**Figure 2:**
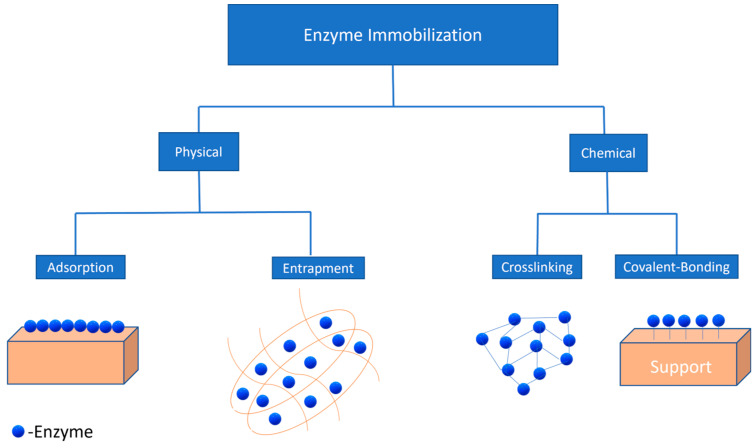
Physical and chemical methods for enzyme immobilization.

Over the last five decades, enzyme immobilization has been used to enhance the ability of enzymes to catalyse reactions in a controlled manner. Immobilization of enzymes has allowed enhanced reusability of enzymes in order to reduce the overall cost of the enrichment process. Different immobilization strategies used to date are mentioned below (Figure 2).

### 6.1. Physical Methods

Physical methods for enzyme immobilization consist of two strategies, physical adsorption and entrapment. In the former, enzyme adsorption occurs on a support material (Figure 2) through weak forces such as hydrophobic interactions, ionic bonding, and Van der Waals forces [120]. Hydrophobic supports, such as octyl-sepharose, have been used to immobilize lipases via physical adsorption, which were further used for the hydrolysis of sardine oil [121]. Moreover, it has been reported that ionic supports such as carboxymethyl and sulfopropyl derivatives tested with immobilizing lipases had better selectivity towards EPA and DHA during fish oil hydrolysis [122]. On the other hand, the latter entrapment technique involves the confinement of the enzyme into a matrix without any chemical reaction, which can reduce the distortion in the structure of the enzyme, affecting the lipase activity [123]. Various sol–gel formulations such as tetramethoxysila, methyltrimethoxysila, and ethytrimethoxysila can also be used for encapsulating lipases and the hydrolysis of oil substrates such as olive oil [124].

New encapsulation approaches such as electrospinning and metal–organic frameworks (MOFs) have also been employed for immobilizing lipases. Recently, lipase from *Burkholderia cepacia* was immobilized via double-needle electrospinning and the gelation approach, collectively, by utilising hydrogel fibre–hydrophobic fibre hybrid membranes (hg-HMs), and showed elevation in the specific activity compared to free enzyme [125]. Moreover, lipase immobilization using Zeolitic imidazolate frameworks (ZIF), a type of MOF, was also used to immobilize lipases from genetically modified *Thermomyces lanuginose*, which was demonstrated to have better catalytic activity [126]. However, electrospinning and MOFs have not been investigated in lipase immobilization for the enrichment of omega-3. The most important drawback of physical methods is the ease of disintegration of the enzymes from the support material, leading to enzyme leaching and the requirement for more enzymes.

### 6.2. Chemical Methods

Chemical methods are further classified into two categories: crosslinking and covalent bonding (Figure 2). Crosslinking is the technique that uses crosslinking agents such as glutaraldehyde in order to improve the interaction between the enzyme and support material. Different support materials such as polyolefin [127] and chitosan-chitin [128] have been used to immobilize enzymes by crosslinking. In one study, crosslinking of *C. rugosa* lipases was carried out using glutaraldehyde, and it was found that the enantioselectivity of the crosslinked enzyme had improved when used for the hydrolysis of olive oil [129]. However, there are various limitations in this technique, such as poor mechanical stability and inefficient reproducibility, which make it less desirable for lipase immobilization.

On the other hand, covalent bonding has been proven to provide better mechanical stability with support that extremely reduces the chances of enzyme leaching into the media and also allows the reactivation of enzymes [130]; moreover, through covalent binding immobilization, the highest enzymatic activity was reported when compared to other methods. Various support materials were used for immobilizing enzymes through covalent bonding, as shown in Table 4.

#### Covalent Bonding-Based Immobilization of Lipase for the Enrichment of Omega-3 PUFAs

Covalent bonding is highly efficient in terms of enzyme stability due to its strong interaction with the support, which reduces enzyme loss. This valuable characteristic of covalent bonding has led to an increased number of studies carried out, which are associated with the immobilization of lipase through covalent bonding (Table 4). The amination of lipases from *Geotrichum candidum* was carried out to facilitate covalent bonding with carboxymethyl and sulfopropyl agarose beads for efficient immobilization, and it was found that the immobilized enzyme was stable up to two cycles and its overall stability had increased by 3.2-fold in hydrolysis compared to the free aminated enzyme [131]. Furthermore, epoxy-functionalised silica particles were also used for immobilizing lipases from *Rhizomucor miehei* and it was reported that the immobilized enzyme can be reused up to five cycles, where it was able to enrich EPA and DHA content with a ratio of 6.8:1, which was higher compared to the free enzyme [132]. In 2019, ferrous nanoparticles were used for easier recyclability of the enzyme through magnetic separation. The research concluded that the immobilized enzyme had shown around 65% activity up to seven reuse cycles and had better thermostability, even at higher temperatures of 95 °C [133].

Moreover, the application of an ecofriendly support matrix for immobilizing enzymes is also gaining interest as it can assist in mitigating various environmental problems. For instance, silica from agricultural waste such as rice husk was utilised to create amino-functionalised nano–silica systems that were used to immobilize lipases derived from *Candida rugosa* through covalent bonding. A 2-fold increase in relative enzyme activity was obtained at 45 °C and a 2.5-fold increase in EPA content was reported using this immobilized enzyme for the hydrolysis of oil derived from *N. oceanica* [134]. In a recent study, crosslinker azelaic acid was employed, which allowed the covalent bond between lipase molecules to form crosslinked enzymes, and a selectivity ratio of 22 was obtained for EPA and DHA that retained activity up to 60% for five cycles [135]. Thus, covalent bonding-based immobilization can be ascribed as a promising method for enzyme immobilization; however, further studies are required for the immobilization of lipases generated from marine-derived sources that can support their claim of being suitable lipases for pharmaceutical and nutraceutical applications.

## 7. Concluding Remarks and Future Directions

The amount of research carried out in the area of omega-3 enrichment clearly suggests the importance of PUFAs in the medical and commercial sectors. Lipases are enzymes able to enrich omega-3 from different substrates such as fish oils and algal oils. Lipases from marine microorganisms hold an advantage due to their unique properties such as thermostability and tolerance against organic solvents; however, there is always a risk of loss in stability and activity of lipases over time. The method of covalent bonding-based immobilization of lipases to various support materials is the current solution that is applied to overcome this problem; however, the support materials used should have a minimal negative impact on the environment. Thus, greener and more inexpensive options for this method need to be investigated, such as the immobilization of lipases on plasma-polymerised surfaces as it does not demand the use of any organic solvents during the process. Furthermore, omega-3-enriched concentrates generated from marine microorganism lipases—that will further be used in food fortification—need to be investigated for their effects on the human gut microbiome as it can be helpful to understand the biochemical route through which omega-3 can infer positive effects on human health. Nevertheless, there are very limited studies carried out using oil from sources such as microalgae to meet the demand for essential omega-3 concentrates. Microalgae can be considered a suitable and sustainable oil source as it falls under the category of GRAS, can be grown in a controlled manner, and various species of microalgae such as thraustochytrids have been reported with higher amounts of EPA and DHA content. In this way, enriched omega-3 concentrates can also sustain the market demand for populations that follow a vegetarian diet. In summary, further development and studies are required to improve enzymatic enrichment using cost-effective and sustainable approaches and to divert the focus towards using oils from sources that can be consumed by a larger number of the population.

## Figures and Tables

**Table 1:** Lipase-producing microorganisms and their lipolytic activity.

Organism	Name	Lipolytic Activity (Unit per Gram)	References
Yeast	*Candida intermedia*	42.0 ^a^	[54,55,56]
	*Pichia guilliermondi*	43.6 ^a^	[54,57]
	*Candida parapsilosis*	10.4 ^a^	[54,56,58]
	*Lodderomyces elongisporus*	16.6 ^a^	[54,59,60]
	*Candida rugosa*	26.9 ^a^	[54,61,62]
	*Candida antartica*	18.52 ^c^	[63]
	*Yarrowia lipolytica*	5.1 ^a^	[54,64,65]
	*Rhhodotorula mucilagionosa*	4.0 ^a^	[54]
Bacteria	*Marinobacter hydrocarbonoclasticus*	4.15 ^b^	[66]
	*Oceanobacillus caeni*	58.84 ^b^	[67]
	*Bacillus sonorensis*	14.17 ^b^	[68,69]
	*Bacillus cereus*	55.17 ^b^	[70,71]
	*Halomonas aquamarina*	4.20 ^b^	[66]
Microalgae	*Nannochloropsis oceanica*	18.3 ^d^ unit/L	[72]
	*Botryococcus sudeticus*	36.6 ^b^	[73]

^a^: units per gram of cell dry weight, ^b^: units per millilitre, ^c^: unit per milligram, ^d^: units per litre.

**Table 2:** Omega-3 PUFA-enrichment techniques.

Enrichment Method	Principle	Omega-3 Enrichment Content	Limitations	Ref.
Urea precipitation	Complex formation of urea with MUFAs	45–60%	Use of flammable solvents, urea and FA complex disposal	[99]
Supercritical fluid extraction	Selective separation ability of SC-CO_2_	60%	High cost	[100]
Molecular distillation	Differences in molecular weight and boiling point	60%	High temperature required	[101]
Enzymatic	Selective hydrolysis	70%	Loss of stability and activity of enzyme	[102]

MUFA: monounsaturated fatty acids; SC-CO_2_: supercritical carbon dioxide; FA: fatty acid; Ref.: References.

**Table 3:** Lipases derived from marine microorganisms and their PUFA-enrichment ability using different oil substrates.

Lipase Source	Lipase Type	Oil Substrate	PUFA Enrichment Content (Initial)	Reference
*Candida cylindracea*	AY “Amano” 400SD	Tuna oil	57.7% (34.3%)	[113]
	AY “Amano” 30SD		Less than 50% (34.3%)	
	AY “Amano” S		Less than 50% (34.3%)	
*Rhizopus oryzea*	DF “Amano” 15		Less than 50% (34.3%)	
Aspergillus oryzae	G “Amano” 50		Less than 50% (34.3%)	
*Candida antarctica*	Lipozyme 435		Less than 50% (34.3%)	
*T. lanuginosus/A. oryzae*	Lipozyme TL 100 L	*Nannochloropsis* species	70 % (48.5%)	[11]
*T. lanuginosus/F. oxysporum/A. oryzae*	Lecitase^®^ Ultra		53.9% (48.5%)	
*R. oryzae*	Lipase D		55.9% (48.5%)	
*Candida rugosa*	Extracellular lipase	Skipjack tuna eyeball oil	30% (27%)	[114]
*Pseudomonas fluorescens*	Recombinant	Fish oil	72.8% (43.16%)	[115]
*Candida antarctica B*	CALB, Novozym^®^ 435	Pure EPA and DHA concentrate	0.75% (16%)	[116]
*Candida antarctica B*	CALB, Novozym^®^ 435	Crude fish oil	74.6% (27.57%)	[117]
*Candida antarctica B*	CALB, Novozym^®^ 435	Cobia liver oil	94% yield	[118]

**Table 4:** Different materials used for covalent bonding of lipases for immobilization with a comparison of stability, activity, reusability, and omega-3 enrichment as compared to free enzyme form.

Material Used	Lipase Source	Activity and Stability (Compared to Free Enzyme)	Reusability	Omega-3 Enrichment Ability	Reference
Carboxymethyl and sulfopropyl agarose beads	*Geotrichum candidum*	10-fold stability retained at 50 °C	Stable up to 2 cycles	3.2-fold increase in hydrolysis	[131]
Silica Epoxy	*Rhizomucor miehei*	25% increase in activity at 50 °C	Stable up to 5 cycles	6.8:1 (released EPA–released DHA)	[132]
Magnetic nanoparticles	Recombinant *Bacillus subtilis*	10% increase in activity at 95 °C	Stable up to 7 cycles	1.5 times higher DHA selectivity	[133]
Nanoparticles Nano–silica system	*Candida rugosa*	2-fold increase in activity at 45 °C	Not mentioned	2.5-fold EPA enrichment	[134]
Crosslinked enzyme (CLE)	*Thermomyces* *lanuginosus*	60% activity was retained at 25 °C	Stable up to 5 cycles	22:1 (released EPA–released DHA)	[135]

## Data Availability

Data are contained within the article.
